# Combined extract of heated TC1, a heat-killed preparation of *Lactobacillus* *casei* and alpha-galactosyl ceramide in a mouse model of cervical cancer

**DOI:** 10.1186/s13027-022-00464-w

**Published:** 2022-09-20

**Authors:** Dorsa Haghighi, Shaghayegh Yazdani, Mahdieh Farzanehpour, Hadi Esmaeili Gouvarchinghaleh

**Affiliations:** 1 Department of Microbiology, Faculty of Advanced Science and Technology, Tehran Medical Sciences, Islamic Azad University, Tehran, Iran; 2 Applied Virology Research Center, Baqiyatallah University of Medical Sciences, Tehran, Iran; 3 Department of Microbiology, Faculty of Medicine, Tehran Medical Sciences, Islamic Azad University, Tehran, Iran

**Keywords:** TC1 cell line, Cervical cancer, *Lactobacillus casei*, Alpha galactosyl ceramide, Immunotherapy

## Abstract

**Background:**

Nowadays, cancer is the leading cause of death among threats to humanity, necessitating prompt action and preparation. Cervical cancer is one of the most common cancers in women and is currently treated with surgery, radiation, chemotherapy, and immunotherapy, among other treatments. Current oncology approaches focused on the simultaneous development of safe and effective cancer multi-agent therapies. The present study aimed to evaluate the effects of a combined extracts of heated TC1, a heat-killed preparation of *Lactobacillus* *casei*, and alpha-galactosyl ceramide (α-GalCer) in a mouse model of cervical cancer.

**Material and methods:**

Cervical cancer in the mouse model was prepared by TC1 cells subcutaneous injection into the left flank of female C57BL/6 mouse aged 6–8 weeks (n = 80). After the appearance of the palpable tumor, the mice with cervical cancer were randomly devoted to 8 (ten-member) groups. The mice in some groups were treated with PBS, TC1 cell extract, *L. casei* extract, α-GalCer, and a combination of the mentioned treatments. Then, they were evaluated the splenocytes proliferation, lactate dehydrogenase production and nitric oxide. Moreover, IL-4, IFN-γ, and TGF-β cytokine levels of splenocytes supernatant the mice were measured. In all evaluations, a statistical difference of less than 0.05 (*P* ˂ 0.05) was considered as a significant level.

**Result:**

The findings revealed that the combination therapy group (heated TC1 cell and *L. casei* extracts with α-GalCer) significantly increases the splenocytes proliferation (MTT) (0.358 ± 0.04 OD), LDH production (45.9 ± 2.3 U/L), NO rate (38.4 ± 2.8 µM), and IFN-γ cytokine level (46.6 ± 3.7 pg/ml) (*P* < 0.05). Also, observes a significantly reduces the production of IL-4 (11.6 ± 2.5 pg/ml) and TGF-β cytokines levels (7.8 ± 2.5 pg/ml) (*P* < 0.05) in comparison to the control group.

**Conclusion:**

The study showed that combination therapy of *L. casei* and α-GalCer is an efficient treatment for cervical cancer in the mouse model.

## Introduction

The Human Papillomavirus (HPV) is double-stranded DNA virus, has over 200 various identified genotypes. The infection is assuming the most prevalent sexually transmitted infection (STI), and causes a great number of disorders, including benign lesions (Condylomata acuminata) and pre-malignant lesions and different cancers [1]. Cervical cancer is one of the top five common neoplasms among women all over the world [2]. All women face the risk of cervical cancer. It is common in women of over 30. Chronic infection with some kinds of HPV is the main reason for cervical cancer. HPV is a common virus transmitted during intercourse. It is present in most sexually active people; however, few women are affected by cervical cancer [3]. The TC1 cervical carcinoma can be applied as an experimental animal tumor cell model for human cervical cancer, because it is highly transplantable, tumorigenic, and invasive. Comparing with most tumor models, TC1 cell lines can concurrently metastasize from the primary tumor in the cervical gland to other sites such as blood, liver, brain, lung, bone and lymph nodes just, similar to human cervical cancer [4]. Recent studies show that alpha galactosyl ceramide, glycosphingolipid derived from a sea sponge known as a combination with anti-metastasis, antitumor and immune system stimulates the capability of α-GalCer and increase the proliferation and activity of NK and NKT cells and production of cytokines [5]. Combination therapy with Fluorouracil (5-FU) and alpha galactosyl ceramide in mouse model of liver tumor increased the expression of NK activating molecules on cancer cells, which had a synergistic antitumor effect on liver tumors [6]. Probiotics are organisms believed to improve health conditions. *Lactobacillus casei* is one of the *lactobacillus* bacteria considered a safe probiotic [7, 8]. Being anticancer is the most crucial property of probiotic bacteria, along with some other properties [8]. It was also proved that some strains, including *L. casei* might modify immune responses against solid tumors when used orally [9, 10]. For instance, in the reports the regular *L. casei* intake can improve the cytotoxicity of the natural killer cells and adoptive immune responses in rats bearing invasive ductal carcinoma. Moreover, the complementary potential of a killed preparation of *L. casei* was investigated in early surveys [11]. However, there is little data on the possible complementary benefits of the killed preparation of *L. casei* in a tumor vaccine. Current oncology approaches focused on the simultaneous development of safe and effective cancer multi-agent therapies. For this reason, the present study aimed to evaluate the effects of a combined extracts of heated TC1, a heat-killed preparation of *L. casei*, and α-GalCer in a mouse model of cervical cancer.

## Materials and methods

### Reagents

The 3-(4,5-Dimethylthiazol-2-yl)-2,5-diphenyltetrazolium bromide (MTT), Griess Reagent Kit, Dulbecco's Modified Eagle Medium (DMEM), Fetal Bovine Serum (FBS), Dimethyl sulfoxide (DMSO), and Phosphate-buffered saline (PBS) were acquired from Kalazist (Tehran, Iran). From Karmania Pars Gene (Kerman, Iran), ELISA kits for enzyme-linked immunosorbent assays (ELISA) were ordered.

### Cell culture

**Fig. 1:**
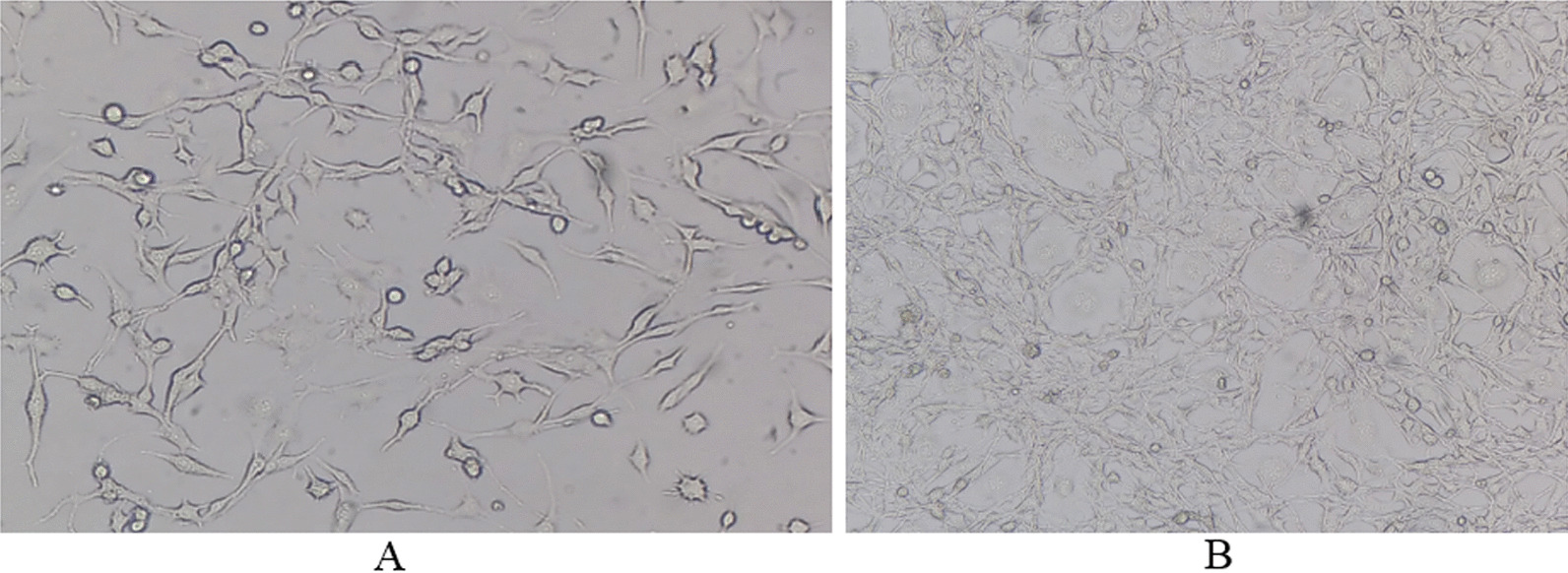
TC1 cell line growth under cell culture condition. **A** 3 days after cell culture, **B** 7 days after cell culture

We received TC1 cells from the Baqiyatallah University of Medical Sciences of Iran. The cells were maintained in monolayer cultures in DMEM supplemented with 10% FBS and cultivated at 37 °C in a humidified atmosphere with 5% CO2 (Fig. 1) [12].

### Bacterial strains and growth conditions

*L. casei* (ATCC:393) was purchased from the central laboratory of Baqiyatallah University of Medical Sciences of Iran. A Rogosa’s medium was applied to cultivate the bacteria. The process was performed at 37 °C for 24 h [13].

### Preparation of cell and bacterial extracts

In order to obtain cell extracts, 10^4^ TC1 cells were exposed to non-lethal excessive heat (43 °C, 30 min), and freeze-and-thaw (− 196 °C, 30 min). The cells were then centrifuged and utilized as a killed cell extract in the treatment of cancerous mice. Additionally, 3 × 10^8^ CFU/ml of grown bacteria were heated at 56 °C for 60 min in order to obtain the bacterial extract, which was subsequently centrifuged for use in the current investigation [13].

### Experimental design, mice and tumor induction

**Fig. 2:**
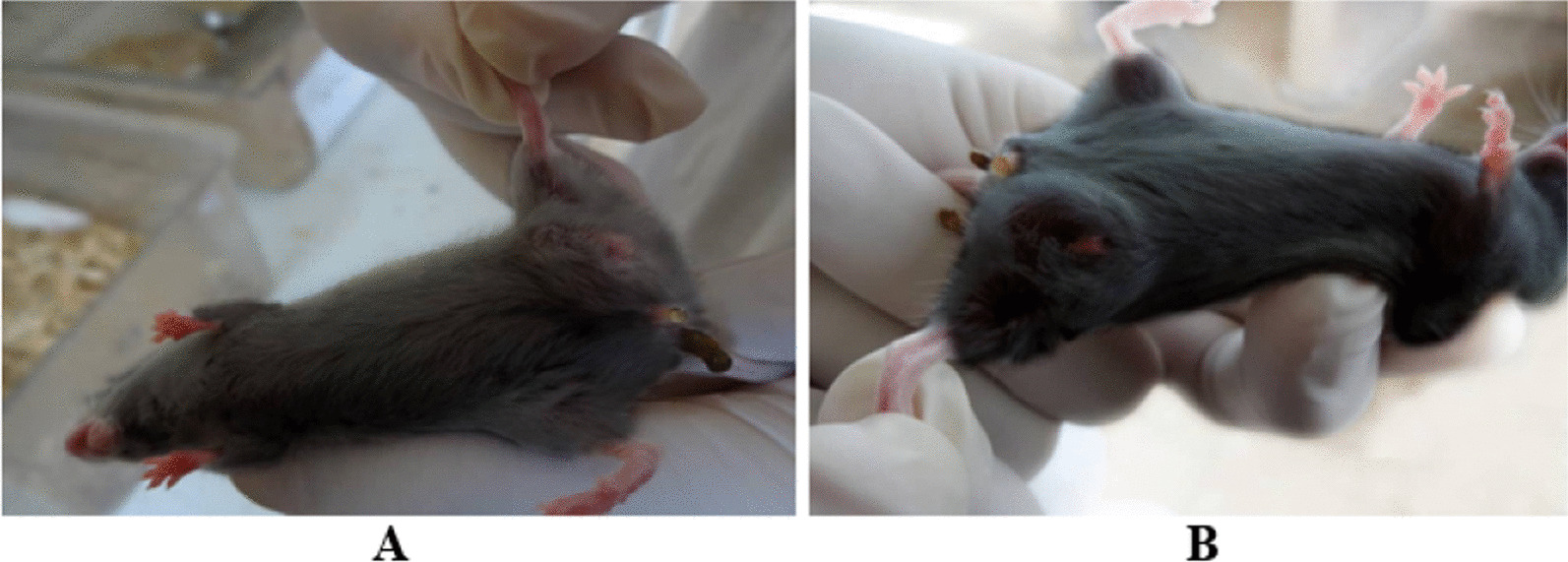
Tumor mice model with palpable tumor. **A** 18 days after injection, **B** 28 days after injection

From Iran's Baqiyatallah University of Medical Sciences, 80 C57BL/6 female mice aged 6 to 8 weeks were purchased. Standard housing for the mice included a temperature range of 22–24 °C, 12-h light/dark cycles, and regular food and water. Before the experiments, all the mice were given a week to get used to their surroundings, and then 1 × 10^5^ live tumor cells were subcutaneously injected into their left flanks in 200 mL of PBS containing 0.2% BSA (Fig. 2) [12]. After that, mice were separated into eight equal groups at random (Table 1). To assess the immunomodulatory effects of Gardasil, the vaccine was given to one group as a positive control group. All groups received treatment in 100 µl volume and twice a one-week interval. One week after the last agent-therapy sampling was started.

**Table 1:** The characteristics of the studied groups

Group	Characteristics (in 100 µL of PBS)
Control	PBS
TC1	10^4^ heated TC1 cells
*L. casei*	3 × 10^8^ CFU/mL of Heated *Lactobacillus casei*
α-GalCer	5 µg of Alpha galactosyl ceramide
TC1 + *L. casei*	Combined of Heated TC1 and Heated *Lactobacillus casei*
TC1 + α-GalCer	Combined of Heated TC1 and α-GalCer
TC1 + *L. casei* + *α-GalCer*	Combined of Heated TC1 and *Lactobacillus casei* extracts and α-GalCer
Gardasil	50 µL of Gardasil vaccine

### The proliferation level of splenocytes

In order to assess the level of splenocytes proliferation, the MTT assay was performed. The splenocytes were placed in 96-well flat-bottomed plates with DMEM media supplemented with 10% FBS (1 × 10^5^ cells/100 μl/well), and they were stimulated with antigens produced from the tumor cells by freezing and thawing (20 μg/ml). The cultures were pulsed with 20 μl of the MTT solution (5 mg/mL) for 4 h at 37 °C after 72 h of incubation. The formazan crystal was then broken down by adding 100 mL of DMSO and vigorously shaking the mixture. Using an ELISA reader, the optical density (OD) at 492 nm was calculated (Dynatech, Denkendorf, Germany). The tests were carried out in sets of three [13].

### Lactate dehydrogenase assay

A LDH detection kit was used to examine cytotoxic activities. This assay is a practical, quick colorimetric method for quantifying cytotoxicity by monitoring the activity of LDH produced from injured cells. Most cells contain the cytoplasmic enzyme LDH, which is stable. The TC1 cell line was utilized as the target cells, and the splenocytes served as the effector cells. The assay environment, DMEM with 1% FBS, was used to wash the effector and target cells before they were co-cultured in 96-well round-bottomed plates for six hours at 37 °C at a ratio of 50 effector cells to one target cell. The plates were then centrifuged, and the supernatants were then deposited on 96-well flat-bottomed plates. The LDH detection mixture was then added to each well and placed in the refrigerator at room temperature for 30 min. An ELISA reader from Dynatech, Denkendorf, Germany, was used to measure the OD at 492 nm [13].

### Measurement of NO in splenocytes population

Using the Griess reagent to measure the nitrite content of the splenocytes culture supernatants, the potential for NO generation was evaluated. After the splenocytes had been cultivated, 50 ml of the cell-free supernatants were taken and combined with 50 ml of Griess reagent, which contains 0.1% sulfanilamide, 3% phosphoric acid, and 0.1% N-(1-Naphthyl) ethylenediamine. The resulting combination was left to sit at room temperature in the dark for ten minutes. After incubation, an ELISA reader (Dynatech, Denkendorf, Germany) detects the absorbance at 492 nm [13].

### Cytokine assay

One week after the last agent-therapy, mice were euthanized to measure the cytokine assay induced in splenocytes. In an aseptic setting, splenocytes were separated from the mice, single-cell suspensions of the splenocytes were made in DMEM medium containing 10% FBS, and RBCs were eliminated using ACK (Ammonium-Chloride-Potassium) Lysing Buffer. After that, 24-well plates were treated with the cell suspensions (2 × 10^6^ cells/mL), and tumor antigens were pulsed into the plates. These antigens were derived from the tumor cells by freezing and thawing (20 μl). As previously mentioned, tumor antigen was created. Following 72 h, the culture supernatants were gathered. According to the manufacturer's instructions, the ELISA kit was used to measure the production of IFN-γ, IL-4, and TGF-β [13].

### Statistical analysis

The statistical analysis was conducted by using SPSS Statistics 23 and the Tukey's Test. The results are shown as means ± SD. *P* < 0.05 was considered statistically significant.

## Results

### Proliferation level of splenocytes

**Fig. 3:**
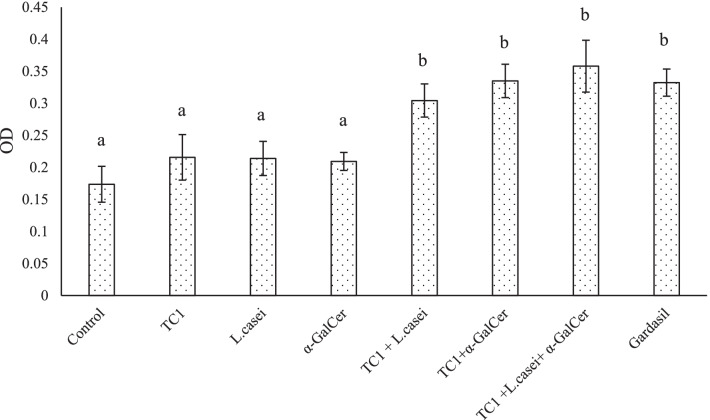
Effects of single and combined treatment on the proliferation level (Optical Density) of splenocytes. The values were normalized. Significant statistical differences between groups in each index are shown by the different superscript letter (*P* < 0.05)

Splenocytes were cultured in the presence of TC1 antigen. The cell proliferation was measured by MTT assay. Splenocytes proliferation in single-factor treatment groups was not significantly different from that in the control group (*P*˃0.05). However, splenocytes proliferation increased significantly in the combination therapy and Gardasil groups in compared to the control group (*P* ˂ 0.05) (Fig. 3).

### Nitric oxide and lactate dehydrogenase production rate

**Fig. 4:**
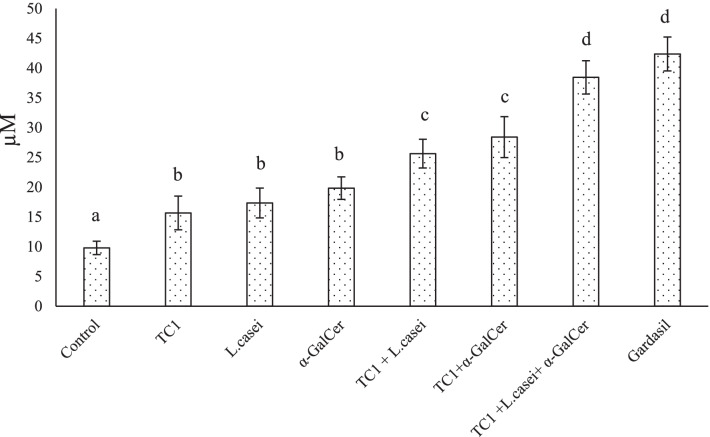
Effects of single and combined treatment, on the NO production of splenocytes. The values were normalized. Significant statistical differences between groups in each index are shown by the different superscript letter (*P* < 0.05)

**Fig. 5:**
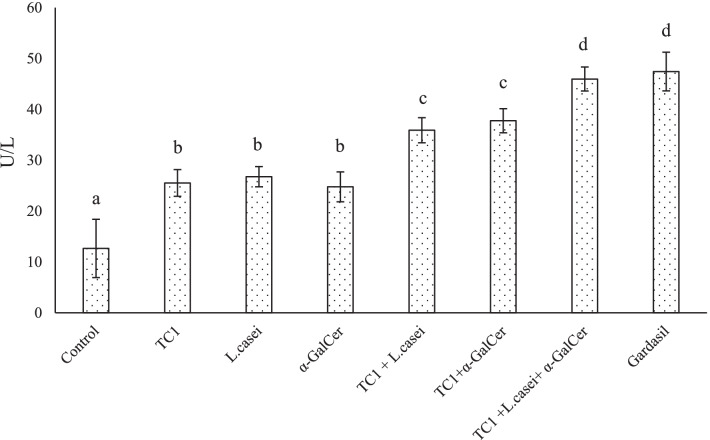
Effects of single and combined treatment, on the LDH production of splenocytes. The values were normalized. Significant statistical differences between groups in each index are shown by the different superscript letter (*P* < 0.05)

The findings revealed that the amount of nitric oxide and lactate dehydrogenase production in single-factor treatment groups increased significantly in comparison to the control group (*P* < 0.05). The same is true, for the combination therapy groups (*P* < 0.05). The highest amount of nitric oxide and lactate dehydrogenase production belonged to the combination treatment group with TC1 extract, bacterial extract and α-GalCer (Figs. 4, 5).

### Splenocytes supernatant cytokines

The findings of cytokine production revealed that the level of IFN-γ production (Table 2) in all single-factor and multi-factor treatment groups had a significant increase relative to the control group (*P* < 0.05). The highest production amount was in the three-factor combination therapy and the Gardasil groups. The results of IL-4 production also showed that all single and multifactorial treatment groups except TC1 cell extract group had a significant decrease in comparison to the control group (*P* < 0.05) (Table 2). The level of TGF-β production decrease significantly just in the group treated with bacterial extract (*P* < 0.05) (Table 2). And all multifactorial treatment groups revealed a significant decrease in compared to the control group (*P* < 0.05).

**Table 2:** Effects of single and combined treatment, on the cytokine production of splenocytes IFN-γ, IL-4 and TGF-β

Groups	IFN-γ	IL-4	TGF-β
Control	5.3 ± 1.9^a^	24.6 ± 2.1^a^	15.2 ± 1.6^a^
TC1	13.1 ± 3.5^b^	22.7 ± 2.8^a^	13.9 ± 0.6^a^
*L. casei*	17.7 ± 2.0^b^	15.7 ± 2.6^b^	12.0 ± 1.3^b^
α-GalCer	15.5 ± 2.2^b^	15.9 ± 1.9^b^	13.5 ± 2.0^ab^
TC1 + *L. casei*	36.1 ± 2.7^c^	12.0 ± 1.4^c^	9.1 ± 1.4^c^
TC1 + α-GalCer	33.2 ± 3.7^c^	11.0 ± 1.7^c^	9.8 ± 1.2^c^
TC1 + *L. casei* + *α-GalCer*	46.6 ± 3.7^d^	11.6 ± 2.5^c^	7.8 ± 2.5^c^
Gardasil	47.1 ± 2.1^d^	12.3 ± 1.6^c^	8 ± 1.5^c^

The values were normalized (Significant statistical differences among groups in each index are shown by the different superscript letter *P* < 0.05)

## Discussion

Overgrowth of cells is called cancer. Since uncontrolled cell proliferation and programmed resistance to death are the main characteristics of cancer cells, what causes the death of cancer cells it may be considered as anticancer agents. Resistance to chemotherapy has been a major problem in recent decades [14]. The use of multi-factor combination therapies has attracted the attention of researchers. In the present study, we used two agents, *L. casei* (probiotic) and α-GalCer (NKT cell stimulator) in treatment of mouse model of cervical cancer. Our findings revealed that the combination therapy group (TC1 cell and *L. casei* extracts, α-GalCer) significantly increases the splenocytes proliferation, lactate dehydrogenase production, nitric oxide and interferon-gamma (*P* < 0.05); and significantly reduces the production of IL-4 and TGF-β cytokines (*P* < 0.05) in comparison to the control. Nutritional compounds and their relationship with human health are of great importance. Probiotics as non-pathogenic microorganisms are present in the digestive system of humans and have beneficial effects on the hosts. Consumption of probiotics leads to the production of a wide range of fermentation products such as high concentrations of short-chain fatty acids [15]. Among all probiotics, bacteria of the *lactobacillus* family such as *lactobacillus acidophilus*, *L. casei* and *Lactobacillus delbrueckii are* the most efficient components of the normal intestinal flora among humans and animals. The role of *lactobacillus* probiotics in facilitating the treatment of colorectal cancer is known for this reason, many studies have been focused currently on investigating the cytotoxic effects of probiotic bacteria [16]. NKT cells are innate immune components restricted to the CD1d receptor and possess the simultaneous characteristics of the T cells and NK cells. INKT cells are the main group of NKT cells in humans and mice. They express the Vα24-Jα18 and Vα14-Jα18 TCRα chains in humans and mice, respectively. Activated iNKT cells rapidly secrete both the cytokines Th1 and Th2, and activate NK cells and other immune cells to stimulate antitumor immune responses [17]. α-GalCer as the main receptor ligand for iNKT cells, is a sphingolipid. It was firstly isolated from *Agelas mauritianas* sponge in 1994, using chloroform and HPLC purification techniques. Many studies have identified α-GalCer as a non-specific (innate) immune system stimulant. The literature shows that the antitumor properties of α-GalCer are mediated by CD1d-bound iNKT cells [18]. Therefore, α-GalCer is applied due to the stimulation of the innate immune arm, especially iNKT. Abdolalipur et al. (2020) showed that levels of IFN-γ, IL-4 and IL-12 after treatment with GM-CSF with *L. casei* probiotic were significantly higher than those in other groups. Also, the apoptosis-inducing ligand associated with intra-tumor TNF-α increased significantly. Furthermore, tumor analysis showed that probiotic group therapy reduced IL-10 accumulation in the tumor microenvironment of the treated mice. Additionally, tumor volume analysis revealed that probiotic group therapy suppressed tumor growth. Abdolalipour et al. (2020) demonstrated that combining GM-CSF and probiotics suppresses HPV-related tumors and enhances specific antitumor immune responses [19]. Jacouton et al. (2019) determined the systemic role of T cells in protecting tumors through a negative correlation of tumor size, T cell subpopulations and increased levels of Foxp3 in tumor-bearing mice. And lastly, a negative relationship between tumor size and NK cells, local migration of NK cells and cytotoxic activity specific for BL23 treatment were observed. Studies showed that the IL-2 signaling pathway has a crucial role in antitumor effects of the probiotic strain *L. casei* BL23. They also suggest more research on probiotic strains for therapeutic applications in clinical practice, specifically for colorectal cancer treatment [10]. Jafari et al. (2017) suggested that animals receiving probiotics face better survival curves and tumor growth rates than tumor-bearing mice and negatively controlled mice. Immunization considerably enhances nitric oxide production and cytotoxicity of natural killer cells in spleen cell culture of tumor-bearing rats. Moreover, immunotherapy increases IFN-γ secretion, while it decreases IL-4 and TGF-β secretion in the splenocytes population in comparison to the splenocytes from other groups. Combined immunotherapy with heated 4T1 cells and heated *L. casei* also leads to useful results in the mice breast cancer model [13]. Yazdi et al. (2009) showed that *L. casei* oral administration could inhibit tumor growth and increase local inflammation in DTH assays due to increase the efficiency of immune responses. In addition, oral administration of *L. casei* may regulate immune responses and upset the Th1 balance and may be efficient for cancer immunotherapy; however, further studies are necessary to investigate other mechanisms of this effect [20]. Soltan Dallal et al. (2015) showed that *lactobacillus* supernatant reduces cell proliferation and increases cell apoptosis. No meaningful effect on cell necrosis has been reported. On the other hand, *lactobacillus* extract decreases cell proliferation and increases cell apoptosis. *Lactobacillus* extract also leads to cell necrosis. Moreover, supernatants and cell extracts of probiotic agents reduce cell migration and invasion [21]. Zhang et al. (2019) reported that NKT cells are quasi-innate T cells with CD1d restriction that express T cell receptors and NK cell markers. The main group of NKT cells is the same among humans and rats. The most common function of iNKT cells is being potentially antitumor. Since discovery of the function from 25 years ago, the primary ligand of iNKT cells and the, α-GalCer has been used in more than 30 antitumor clinical trials. To realize its therapeutic potential, several preclinical models have been developed in order to optimize α-GalCer-based designs and strategies. However, since there is no standard protocol for α-GalCer [18]. Lee et al. (2016) evaluated the immunomodulatory effect of dead *lactobacillus plantarum* with nano-sized *lactobacillus plantarum* (nLp) size in RAW 264.7 cells and the mouse primary splenocytes. nLp is the dead, shrunk, processed form of *lactobacillus plantarum* nF1, isolated from kimchi (a traditional Korean fermented cabbage) less than 1 µm in size. The nLp treatment was found out to stimulate nitric oxide production in RAW 264.7 macrophages more than in pure live *lactobacillus plantarum*, and the stimulatory properties may have been largely derived from its cell wall. In addition, nLp induced mouse spleen cell proliferation was greater than that of pLp in particular, high-dose proliferation stimulated as much as lipopolysaccharide at 2 μg/ml. In addition, according to the results of cytokine profiles in spleen cells, nLp treatment promoted Th1 (TNF-α, IL-12 -p70) responses instead of Th2 responses (IL-4, IL-5) and increased Th17 response (IL-6, IL-17A). These findings suggest that dead nLp has potential application, as a functional nutrient to enhance the immune response and in particular as a means of inducing Th1 and Th17 immune responses [22]. Gableh et al. (2016) claimed that the use of α-GalCer as an adjuvant in the DNA of the proposed cervical cancer vaccine increased lymphocyte proliferation, IFN-γ and IL-12 cytokine levels, and inhibited tumor growth [23]. Kim et al. (2010) reported that the use of α-GalCer in combination with tumor antigen-stimulated dendritic cells increased the population of anti-tumor cell T lymphocytes and decreased tumor volume in cervical cancer mice [24]. Ando et al. (2015) showed that the use of α-GalCer in the treatment of a mice model of metastatic lung cancer increased the production of cytokine IFN-γ by spleen cells and the population of anti-tumor cell killer T cells [25]. Li et al. (2014) explained that the use of α-GalCer was effective in the treatment of B-cell lymphoma. It increased the production of IFN-γ and IL-2 by splenic cells and improving the specific pathology of B-lymphoma [26]. The advantage of our study over other studies was that our treatment consisted of both probiotic therapy and alpha-galactosyl therapy at the same time, after which we proved that both innate (NKT cell) and acquired immune systems are stimulated. In previous studies, however, most of the acquired immune arm was considered and the treatments were not used simultaneously.

## Conclusion

According to the results of the present study, it can be said that the combined treatment of mouse model of cervical cancer with *L. casei* cells and alpha galactosyl ceramide is useful and effective. In addition, the present study showed that the beneficial effects of this compound may be in part due to the diversion of immune responses from the production of anti-inflammatory cytokines such as IL-4 and TGF-β to pro-inflammatory cytokines such as IFN-γ and stimulation. Also, considering that it increased the rate of nitric oxide as representatives of the innate immune system, it can be concluded that in this type of combination therapy, both arms of the immune system are activated.

## Data Availability

Not applicable.
